# CNV analysis in 169 patients with bladder exstrophy-epispadias complex

**DOI:** 10.1186/s12881-016-0299-x

**Published:** 2016-04-30

**Authors:** Catharina von Lowtzow, Andrea Hofmann, Rong Zhang, Florian Marsch, Anne-Karoline Ebert, Wolfgang Rösch, Raimund Stein, Thomas M. Boemers, Karin Hirsch, Carlo Marcelis, Wouter F. J. Feitz, Alfredo Brusco, Nicola Migone, Massimo Di Grazia, Susanne Moebus, Markus M. Nöthen, Heiko Reutter, Michael Ludwig, Markus Draaken

**Affiliations:** Institute of Human Genetics, University of Bonn, Bonn, Germany; Department of Genomics, Life & Brain Center, Bonn, Germany; Department of Urology and Pediatric Urology, Ulm, Germany; Department of Pediatric Urology, St. Hedwig Hospital Barmherzige Brüder, Regensburg, Germany; Department of Pediatric and Adolescent Urology, University of Mannheim, Mannheim, Germany; Department of Pediatric Surgery and Pediatric Urology, Children’s Hospital of Cologne, Cologne, Germany; Department of Urology, Division of Pediatric Urology, University of Erlangen-Nürnberg, Erlangen, Germany; Department of Human Genetics, Radboud University Medical Centre, Nijmegen, The Netherlands; Pediatric Urology Center, Department of Urology, Radboud University Medical Centre, Nijmegen, The Netherlands; Department of Medical Sciences and Medical Genetics Unit, Città della Salute e della Scienza University Hospital, University of Torino, Torino, Italy; Institute for Maternal and Child Health, IRCCS Burlo Garofalo, Trieste, Italy; Institute of Medical Informatics, Biometry, and Epidemiology, University Hospital of Essen, University Duisburg-Essen, Essen, Germany; Department of Neonatology and Pediatric Intensive Care, University of Bonn, Bonn, Germany; Department of Clinical Chemistry and Clinical Pharmacology, University of Bonn, Sigmund-Freud-Str. 25, Bonn, D-53127 Germany

**Keywords:** Bladder exstrophy-epispadias complex, Copy number variation, Genetic testing, EFNB1

## Abstract

**Background:**

The bladder exstrophy-epispadias complex (BEEC) represents the severe end of the congenital uro-rectal malformation spectrum. Initial studies have implicated rare copy number variations (CNVs), including recurrent duplications of chromosomal region 22q11.21, in BEEC etiology.

**Methods:**

To detect further CNVs, array analysis was performed in 169 BEEC patients. Prior to inclusion, 22q11.21 duplications were excluded using multiplex ligation-dependent probe amplification.

**Results:**

Following the application of stringent filter criteria, seven rare CNVs were identified: *n* = 4, not present in 1307 in-house controls; *n* = 3, frequency of <0.002 in controls. These CNVs ranged from 1 to 6.08 Mb in size. To identify smaller CNVs, relaxed filter criteria used in the detection of previously reported BEEC associated chromosomal regions were applied. This resulted in the identification of six additional rare CNVs: *n* = 4, not present in 1307 in-house controls; *n* = 2, frequency <0.0008 in controls. These CNVs ranged from 0.03–0.08 Mb in size. For 10 of these 13 CNVs, confirmation and segregation analyses were performed (5 of maternal origin; 5 of paternal origin). Interestingly, one female with classic bladder extrophy carried a 1.18 Mb duplication of 22q11.1, a chromosomal region that is associated with cat eye syndrome.

**Conclusions:**

A number of rare CNVs were identified in BEEC patients, and these represent candidates for further evaluation. Rare inherited CNVs may constitute modifiers of, or contributors to, multifactorial BEEC phenotypes.

## Background

The bladder-exstrophy-epispadias complex (BEEC; MIM %600057) represents the severe end of the uro-rectal malformation spectrum, and has a profound impact on continence and sexual and renal functions. The BEEC is an anterior wall midline defect with variable phenotypic expression. The phenotype ranges from epispadias (E) and classic bladder exstrophy (CBE), to the most severe form, cloacal exstrophy (CE). The latter is often referred to as the OEIS complex (omphalocele, exstrophy, imperforate anus, and spinal defects) [1–3]. Around one third of BEEC patients present with associated urological malformations, e.g. ectopic kidney, renal agenesis, and hydronephrosis. BEEC has an overall prevalence of 2.07 in 100,000 live births, and is more common in males [4]. For the specific subtypes, estimated birth prevalences after the inclusion of terminated pregnancies are 1 in 117,000 in males and 1 in 484,000 in females for E; 1 in 37,000 for CBE; and 1 in 200,000 to 1 in 400,000 for CE [2–7]. Although BEEC can occur as part of a complex malformation syndrome, approximately 98.5 % of cases are isolated [4, 8, 9].

Extensive recent research has implicated both inherited and de novo genetic factors in BEEC etiology. These factors include common single nucleotide polymorphisms (SNPs) [10, 11] and rare larger genomic aberrations, such as chromosomal aberrations and copy number variations (CNVs) (Table 1 [12–28]). Genetic risk factors involving larger genomic regions typically show stronger individual effects on disease causation, and are more likely to have a de novo occurrence. The largest systematic array-based genome-wide CNV study of BEEC to date investigated 110 patients, and identified a de novo 0.9 Mb microduplication on chromosome 19p13.12 in a single CBE patient [22]. Two earlier array-based genome-wide CNV studies, which included a total of 102 CBE patients, identified a duplication of 22q11.21 in four individuals [23, 25]. An additional case report described an array-based CNV analysis in a single CBE patient with a duplication of 22q11.21 [26]. Following array-based genome-wide CNV studies, Draaken et al. [24] used a multiplex ligation-dependent probe amplification (MLPA) based approach to perform a regional screen for 22q11.21 duplications in 244 independent BEEC patients. The authors identified four novel duplications of variable size in four unrelated CBE patients.

**Table 1:** Chromosomal aberrations and CNVs reported in BEEC patients

BEEC phenotype	Other anomalies	Aberration/CNV	Size	Reference
CE (OEIS)	Prominent labioscrotal folds, no apparent genital tubercle, midline defect, imperforate anus, left foot anomaly	del 1p36.33	1.25 Mb	12
CE (OEIS)	Microbrachycephaly, large anterior fontanel, cardial septal defects, rib fusion, limb deformity, typical facial features, developmental delay	del 1p36	2.4 Mb	13
CE (OEIS)	Micrognathia, increased nuchal fold thickness, median clefting or soft and hard palate, low-set malformed ears, camptodactyly, hypoplastic nails	del 1q41	?	14
CBE	Agenesis of corpus callosum, congenital heart disease	del 1q	10.4 Mb	15
CBE	-	del 2p15	0.07 Mb	16
CE (OEIS)	Dysmorphic features	del 3q12.2-q13.2	13 Mb	17
CBE	Wolf-Hirschhorn syndrome	del 4p (?)	?	18
CBE	Wolf-Hirschhorn syndrome	del 4p (?)	?	19
E	-	dup 9p	?	20
CE (OEIS)	Axial hypotonia	del 9q34.1-qter	?	21
CBE	-	dup 19p13.12	0.9 Mb	22
CBE	-	dup 22q11.21	2.52–2.59 Mb	23, 25
CBE	-	dup 22q11.21	2.55–2.57 Mb	23, 25
CBE	Hearing impairment, scoliosis	dup 22q11.21	2.48–2.54 Mb	24, 25
CBE	Hearing impairment, mild neuropsychiatric disorder	dup 22q11.21	2.52–2.59 Mb	24, 25
CBE	-	dup 22q11.21	2.52–2.59 Mb	24
CBE	Short stature, delayed psychomotor development	dup 22q11.21	~2.4 Mb	26
CBE	-	dup 22q11.21	0.75–0.83 Mb	24
CBE	-	dup 22q11.21	0.69–0.77 Mb	24
CBE	-	dup 22q11.21	0.40–0.43 Mb	24
CBE	Short stature	del Xp22.12-pter +	19.95 Mb	27
		dup Xq26.3-qter	20.75 Mb	
CE (OEIS)	Secundum atrial septal defect, cyst in right medulla, tracheobronchomalacia	dup 7p15.1 +	0.34 Mb	
		dup 17q21.31-q21.32	0.64 Mb	28
CE (OEIS)	-	dup 5q21.1	0.12 Mb	
		dup 11p15.1	0.11 Mb	
		dup17q21.31-q21.32	0.13 Mb	
		dup 22q11.1	0.39 Mb	
		del Xp22.31	0.06 Mb	28
CE (OEIS)	Vascular malformation of left leg	del 4p15.31	0.14 Mb	
		del 6q21	0.05 Mb	
		dup17p13.2	0.32 Mb	
		dup 18q12.1	0.06 Mb	28
CE (OEIS)	Patent ductus arteriosus, hemiazygos vein	del 7p21.3	0.23 Mb	
		dup17q21.31-q21.32	0.23 Mb	28

The aim of the present study was to detect further BEEC-associated CNVs by performing a state-of-the-art genome-wide single nucleotide polymorphism (SNP)-array based analysis in 169 BEEC patients. Standardized filter criteria for a genome-wide approach were applied. To detect smaller CNVs, we then conducted a high-resolution analysis of genomic regions previously implicated in BEEC phenotypes using relaxed filter criteria.

## Methods

### Patients, controls, and DNA isolation

The present study was part of an ongoing multicenter investigation of the molecular genetic causes of BEEC. In an earlier study, our group performed a regional screen for 22q11.21 duplications in 244 previously unreported BEEC patients using MLPA [24]. For 169 of these 244 patients, the DNA samples were suitable for genome-wide array-based CNV analysis. None of these 169 unrelated patients carried a 22q11.21 duplication. These 169 patients were therefore used as the cohort for the present analyses. Of these 169 patients (E, *n* = 17; CBE, *n* = 126; CE, *n* = 26), 109 were male and 60 were female. Patients were of Central European (*n* = 128); Spanish (*n* = 24); Italian (*n* = 7); Bosnian (*n* = 2); Croatian (*n* = 1); Portuguese (*n* = 1); and Turkish origin (*n* = 6). The patients were recruited by one of four experienced physicians. For 125 patients, DNA from both parents was available. All patients had a negative family history of BEEC. A total of 1,307 population-based controls were drawn from the Heinz Nixdorf Recall Study (HNR) [29]. The study was approved by the ethics committee of the Medical Faculty of the University of Bonn. Written informed consent was obtained from all participants prior to inclusion.

Blood or saliva samples were obtained from patients, the population-based control group, and (when possible) from the parents of the present BEEC patients. Isolation of genomic DNA from blood was carried out using a Chemagic Magnetic Separation Module I (Chemagen, Baesweiler, Germany). Isolation of genomic DNA from saliva samples was carried out using the Oragene DNA Kit (DNA Genotek Inc., Kanata, Canada).

### Array-based molecular karyotyping

For CNV detection, Illumina’s HumanOmniExpress-12 v1.1 microarray (San Diego, California, USA) was used. This comprises 719,665 markers, and has a median marker spacing of 2.1 Kb. The controls were genotyped using Illumina’s HumanOmniExpress-12 v1.0 microarray. The v1.1 and v1.0 microarrays have an overall marker overlap of 99.98 %. A DNA sample was considered to have failed if less than 99 % of the markers were called on the respective microarray.

### CNV analysis

CNVs were predicted using the program QuantiSNP (v2.2, www.well.ox.ac.uk/QuantiSNP/). This program applies the Objective-Bayes Hidden-Markov model [30]. The following quality control (QC) criteria were used to exclude CNVs: (I) log Bayes factor <30; and (II) regions with <5 consecutive aberrant markers. In a subsequent step, samples which still presented with >10 CNVs (twice the standard deviation), or with a standard deviation of the log R ratio of >0.3, were excluded.

In the subsequent analysis, CNVs were excluded if they: (I) covered both equivocal telomeric regions and HLA-loci; (II) presented without gene content; (III) affected segmental duplications only; (IV) had a frequency in the present control cohort of >1 %; or (V) had >10 entries in the Database of Genomic Variants (DGV; http://dgv.tcag.ca/dgv/app/home).

Of the remaining CNVs, only those with a length of >1 Mb were considered. Filtering was performed using the package ‘intervals’, as implemented in R (R: A Language and Environment for Statistical Computing; http://www.R-project.org), and the UCSC Human Genome Browser assembly hg19 (http://genome.ucsc.edu/) [31].

Irrespective of the above filter criteria, a separate analysis was performed for regions/CNVs previously associated with BEEC phenotypes (see Table 1). This analysis included CNVs with a length of <1 Mb.

Following the application of the above mentioned criteria, all remaining CNVs were visually inspected using GenomeStudio genotyping module (v2011.1, www.illumina.com/). Possible candidate genes within these regions were evaluated for their expression in BEEC-relevant tissues during the respective critical embryonic time frame in mice (E9.5–14.5), as indicated in the Mouse Genome Informatics Database (MGI; http://www.informatics.jax.org/). Further information on the function of these candidate genes was obtained from the Uniprot Database (http://www.uniprot.org/), and via an NCBI literature research of PubMed and OMIM (http://www.ncbi.nlm.nih.gov).

### Quantitative polymerase chain reaction (qPCR)

Confirmation of the remaining visually inspected CNVs was carried out using qPCR and SYBR Green or TaqMan. To detect the origin of each CNV, the respective parents were screened. The qPCR was performed on an ABI Prism 7900HT Fast Real-Time PCR System with SYBR Green (Applied Biosystems, Foster City, USA), as described elsewhere [32]. All primer sequences are available upon request. Two CNVs were confirmed by qPCR using TaqMan Copy Number Assay Hs07478160_cn (Applied Biosystem, Foster City, CA, USA), and LightCycler® 480 Instrument II (384 well version; Roche Diagnostics GmbH, Mannheim, Germany). This probe locates at nucleotides chr1:1,398,345-1,398,369 (hg19). Copy numbers were calculated using the ΔΔCt method, as implemented in the CopyCaller Software (v2.0, http://www.appliedbiosystems.com/support/software/copycaller/).

### *EFNB1* sequence analysis

In all 25 female patients with CE, sequence analysis of the *EFNB1* gene was performed. Of these patients, 23 had undergone previous microarray analysis, without detection of any disease-associated CNVs [22, 23]. All five exons with their adjacent splice sites were amplified by PCR (oligonucleotide sequences obtainable on request). For mutational analysis, PCR-amplified DNA products were subjected to direct automated sequencing (3130XL Genetic Analyzer, Applied Biosystems, Foster City, USA) and sequencing was performed for both strands of each amplicon. Nucleotides were numbered according to GenBank entry NM_004429.4.

### Karyotype analysis

Conventional cytogenetic analysis was performed using standard procedures (data not shown).

## Results

QuantiSNP array analysis in the initial 169 samples detected 13,767 putative CNVs. The samples of 18 patients failed to meet initial QC criteria, and were excluded from further analysis.

Using the primary filter criteria, six rare CNVs were identified (Table 2). All six reside in regions not yet implicated in BEEC. These six CNVs comprised five duplications and one deletion, and were identified in a total of seven patients. Examination of CNVs of <1 Mb in regions previously associated with BEEC (Table 1) revealed six additional CNVs in a further six patients (Table 2), and comprised deletions only. For three of these 13 CNVs, confirmation of their presence was impossible due to their partial overlap with segmental duplications.

**Table 2:** Potential disease causing CNVs observed in 169 BEEC patients

Chromosomal band	Position [hg19]	Size [Mb]	Pat	Sex	Phenotype	Aberration	RefSeq genes	Inheritance	Frequency in inhouse controls
CNVs found in regions not previously associated with BEEC									
4q26	4:117,047,226-118,043,617	1.00	5	male	E	duplication	*TRAM1L1, MIR1973*	paternal	0
5q22.2	5:111,778,778-112,842,992	1.06	6	female	CBE	duplication	7 genes, see Results	paternal	0
13q33.1-q33.2	13:104,746,408-106,422,213	1.68	11	male	CBE	deletion	*DAOA, DAOA-AS1, LINC00343*	maternal	0
Xq11.1-q13.1	X:62,038,249-68,117,977	6.08	17	female	CBE	duplication	43 genes (e.g. *EFNB1*)	paternal	0
22q11.1^a^	22:16,114,244-17,294,251	1.18	14	female	CBE	duplication	10 genes, see Results	n. c.	0.0022^b^
Xp22.31	X:6,430,651-8,135,053	1.70	15	female	CBE	duplication	7 genes, see Results	maternal	0.0008^b^
Xp22.31	X:6,436,087-8,135,053	1.70	16	female	CBE	duplication	7 genes, see Results	paternal	0.0008^b^
CNVs in regions previously associated with BEEC									
1p36.33	1:1,385,211-1,425,700	0.04	19	male	CBE	deletion	*ATAD3B, ATAD3C*	paternal	0
1p36.33	1:1,385,211-1,425,700	0.04	20	female	CBE	deletion	*ATAD3B, ATAD3C*	maternal	0
1p36.33	1:1,415,012-1,447,325	0.03	21	male	E	deletion	*ATAD3B*	n. c.	0.0008
1q41	1:216,277,327-216,431,962	0.16	2	male	CBE	deletion	*USH2A*	maternal	0
9q34.2	9:136,128,546-136,133,506	0.01	9	female	CBE	deletion	*ABO*	maternal	0
19q13.42	19:53,932,295-54,010,277	0.08	22	female	CBE	deletion	*ZNF761, ZNF813, TPM3P9*	n. c.	0.0015

^a^CNV resides in a region typically amplified in cat eye syndrome, but karyotype analysis detected no supernumerary marker chromosome; ^b^CNVs not confirmed (n. c.) due to their partial overlap with segmental duplications

The six larger CNVs included a 1.7 Mb duplication comprising seven RefSeq genes, which was detected at Xp22.31 in two CBE females (Pat. 15 and 16). These two identical duplications were of maternal origin (Pat. 15), and paternal origin (Pat. 16) respectively. Since the mother of Patient 15, and the father of Patient 16, were unaffected, it is unlikely that this CNV was a highly penetrant genetic causal factor. However, it may nonetheless contribute to disease development.

Furthermore, we detected a 6.08 Mb duplication at Xq11.1-q13.1 in a CBE female, which had been transmitted from the non-affected father. This female patient also presented with persistent foramen ovale and bilateral inguinal hernia. The duplication represents the largest CNV detected in the present study, and encompasses 43 RefSeq genes. This region contains the *Ephrin B1* (*EFNB1*) gene. *EFNB1* has previously been associated with craniofrontonasal syndrome (MIM #304110), a severe craniofacial midline defect that is only expressed in female carriers. Interestingly, two reports in the literature describe the co-occurrence of CE—the most severe form of the BEEC—and craniofrontonasal syndrome in two unrelated female patients [33]. Therefore, although the female patient with the *EFNB1* comprising duplication displayed CBE and not CE, the subsequent sequence analysis focused on all female CE patients in our cohort (*n* = 25 CE females). Sequence analysis of all five *EFNB1* exons and their adjacent splice sites revealed no mutation in any of these 25 CE females. In one patient, an extremely rare but silent variant was detected in exon 5 (rs143341175, p. Ser281=). No minor allele frequency (MAF) for this variant is given in dbSNP. In four patients, a common polymorphism was detected in the 3′-UTR (rs2230423, C/T, MAF 0.1 in the European population).

**Fig. 1:**
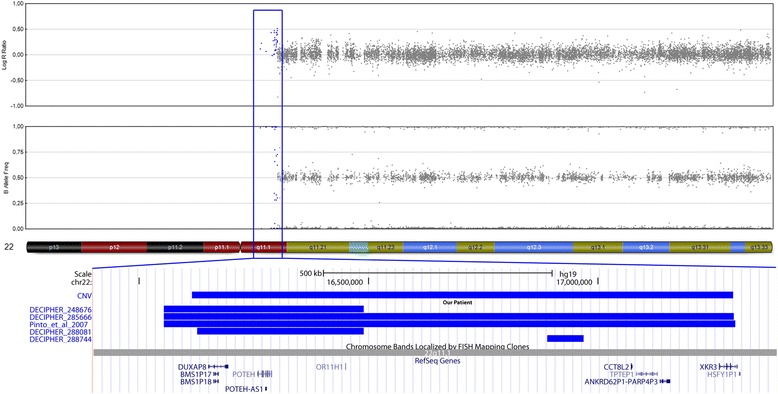
Results of molecular karyotyping: (*Top*) Chromosome 22q11.1 duplication comprising 34 markers (*boxed*), observed in a CBE female as compared to those described in an earlier report [42] and listed in the DECIPHER database. (*Bottom*) RefSeq genes (according to hg19) located in the duplicated region

Interestingly, one female CBE patient who additionally showed coxa valga (Pat. 14), carried a 1.18 Mb duplication on chromosome 22q11.1 (Fig. 1), which involves a region typically amplified in cat eye syndrome (CES; #115470). Karyotype analysis detected no supernumerary marker chromosome. Due to the partial overlap of this CNV with segmental duplications, qPCR could not be performed in the mother. As we did not had a paternal sample, it was not further investigated, whether this CNV had been inherited. The breakpoints did not coincide with the known low copy repeat (LCR) regions, as this CNV is proximal to LCR-A. CES conventional cytogenetic analysis from peripheral blood revealed a normal female karyotype (46,XX) in 30 metaphases. No supernumerary marker chromosome 22 was detected. The region affected by this duplication harbors six pseudogenes, and four genes encoding the transcripts for POTE ankyrin domain family member H (*POTEH*); olfactory receptor 11H1 (*OR11H1*); putative T-complex protein 1 subunit theta-like 2 (*CCT8L2*); and XK-related protein 3 (*XKR3*) (Fig. 1).

In a male with epispadias and penoscrotal transposition (Pat. 5), a 1 Mb duplication was detected at 4q26. This was of paternal origin. This duplication affects the translocation associated membrane protein 1-like 1 (*TRAM1L1*) gene and one microRNA (*MIR1973*). A CBE female (Pat. 6) was found to carry a 1.06 Mb duplication on chromosome region 5q22.2, which involved seven genes. The duplicated genes were adenomatous polyposis coli (*APC*); signal recognition particle 19 kDa (*SRP19*); U2 small nuclear ribonucleoprotein auxiliary factor 35 kDa subunit-related protein 1 (*ZRSR1*); receptor accessory protein 5 (*REEP5*); decapping mRNA 2 (*DCP2*); mutated in colorectal cancers (*MCC*); and testis-specific serine kinase 1B (*TSSK1B*).

Finally, a 1.68 Mb deletion at 13q33.1-q33.2 was detected in a male CBE patient (Pat. 11). This affected the D-amino acid oxidase activator (*DAOA*) gene and its antisense RNA (*DAOA-AS1*), as well as a long intergenic non-coding RNA (*LINC00343*).

In chromosomal regions previously associated with BEEC, we identified six deletions with an unknown effect. A deletion of chromosomal region 1p36.33 was observed in three patients. Two of these patients had CBE (Pat. 19 and 20, Table 2) and these individuals carried the same 40 Kb deletion. Patient 19 was male, and had inherited the CNV from his healthy father. Patient 20 was female, and had inherited the CNV from her healthy mother. A smaller, overlapping 30 Kb 1p36.33 microdeletion was detected in a male patient (Pat. 21) with epispadias. However, confirmation of this microdeletion was impossible due to the presence of a partially overlapping segmental duplication. The larger CNVs of this 1q36.33 region encompass two members of the family of mitochondrial AAA + −ATPase ATAD3 genes, i.e. *ATAD3B* and *ATAD3C.* The smaller CNV affects *ATAD3B* only.

In addition, we detected a small, maternally inherited deletion of chromosomal region 1q41 in a male CBE patient (Pat. 2). This deletion affects the *Usherin* (*USH2A*) gene, which is mutated in mild autosomal recessive usher syndrome 2A.

A maternally transmitted 10 Kb deletion at 9q34.2, which encompasses the ABO blood group gene, was detected in a CBE female (Pat. 9). A further CBE female patient (Pat. 22) carried an 80 kb deletion at 19q13.42. The genes affected by this 19q13.42 deletion encode two zinc finger proteins (*ZNF761* and *ZNF813*), and the tropomyosin 3 pseudogene 9 (*TPM3P9*) non-coding RNA. However, confirmation of this CNV was impossible due to the presence of a partially overlapping segmental duplication.

## Discussion

The largest CNV detected in the present study was a paternally inherited 6.08 Mb duplication. This contains 43 RefSeq genes, and was found in a female CBE patient (Pat. 17) (Table 2). Previous authors have reported that an increased dosage of X-linked genes impacts normal neurocognitive development [34]. The present 6.08 Mb duplication is listed in Decipher and the Database of Genomic Variants (DGV; http://dgv.tcag.ca/) as having shown association in several patients with neurocognitive impairment. In contrast to patients from these previous reports, the present female CBE patient was otherwise healthy and showed no neurocognitive impairment. Of the 43 RefSeq genes within the duplicated region, we considered *EFNB1*, encoding the ephrin receptor ligand ephrin-B1, to be a promising candidate gene. Although heterozygous mutations in this gene cause craniofrontonasal syndrome, the genetic defect causes no—or only mild—abnormalities in male carriers [35]. If a duplication of *EFNB1* exerts a similar effect, this might explain our observations in a healthy carrier father. This hypothesis is supported by the familial hypertelorism study of Babbs et al. [36], which identified a duplication of *EFNB1* in three affected females. A duplication model led to an imbalance in murine Ephrin-B1 expression and abnormal cell sorting. Interestingly, around 10 % of mice—whether heterozygous, homozygous, or hemizygous for the conditional *EfnB1*^Lox^ allele—died within 24 h due to severe cleft palate [36]. The literature also includes at least two reports of female patients with craniofrontonasal syndrome and CE, thus suggesting a common etiology [37, 38]. Moreover, from embryonic day 10.5, *Efnb1* expression has been detected in the renal, urinary, and reproductive systems of the mouse [39]. Research has also shown, that in humans another member of the family of ephrin receptor ligands, ephrin-B2, acts as a signaling molecule in uro-rectal development [40]. However, we detected no potential causal *EFNB1* variant for CE in the present cohort of 25 female patients, although the sample size may have been too small to detect rare causal mutational events. Furthermore, we cannot exclude the possibility that the method applied in the present study overlooked mutations in the promoter region, as-yet-unknown regulatory sequences, or non-coding regions.

In that context of ephrin receptor ligands, Walczak-Sztulpa et al. [41] also reported genital malformations in patients with deletions of 13q33-34, where *EFNB2* is located. The authors suggested, that this chromosomal region harbors a gene for male genital development. Of note, the *EFNB2* gene is directly adjacent to the 13q33.1-q33.2 deletion found in our male patient 11 (Table 2). *EFNB2* has also been analyzed as a candidate gene in 13 patients with OEIS complex in the study by Vlangos et al. [28] however, no mutations were identified. Hence, further studies are warranted to investigate a potential dose effect of *EFNB1* and *EFNB2* in the etiology of BEEC, and to determine whether *EFNB1* shares functions with *EFNB2*.

In previous studies, our group and others have generated strong evidence for the involvement of 22q11.21 duplications in the etiology of BEEC [23–26] and thus, the present finding of a 1.18 Mb duplication of the neighboring chromosomal region 22q11.1 (Fig. 1) in a female CBE patient (Pat. 14) is interesting, particularly since this CNV resides within the region typically amplified in cat eye syndrome (CES; MIM #115470). This duplication has been detected in numerous (apparently healthy) controls [42–44], and may thus represent a benign variant. However, it remains possible that it is causally related to the phenotype but with incomplete penetrance, as has been observed for the duplication 22q11.21 [23–25]. Interestingly, the CNVs on 22q11.1 and Xp22.31 were previously reported in a patient with OEIS complex [28]. Although these CNVs are not identical, the duplicated 22q11.1 region is partially encompassed within the duplicated region in our patient 14, and the region on Xp22.31 is a much smaller deletion that is completely encompassed within the region, that is duplicated in our patients 15 and 16.

Of the six deletions identified in previously implicated (Table 1) chromosomal regions, the deletion of chromosomal region at 1p36.33 was detected in three patients. This region is involved in one of the most common terminal subtelomeric microdeletion syndromes, i.e. the 1p36 contiguous gene deletion syndrome, which typically presents with central nervous system involvement, cardiac defects, and dysmorphic craniofacial features [45]. Two deletions of chromosomal region 1p36 in two different CBE patients involved *ATAD3B* and *ATAD3C*, while in the patient with epispadias only, *ATAD3B* was deleted. Interestingly, de novo deletions affecting this chromosomal band and deleting all three *ATAD3* genes were detected in two previously reported patients with CE, i.e. the severest form of BEEC [12, 13]. However, the patients presented in these separate reports may actually represent one (the same) single patient as the description of the genital phenotype is strikingly similar. Nevertheless, this finding suggests an additive effect of *ATAD3* genes in BEEC etiology. While *Atad* genes are expressed in early embryonic development [46, 47], *Atad3* deficient mice usually die at E7.5, and heterozygotes display no urogenital anomalies [47]. Moreover, around 70 heterozygous deletions, which involve all human *ATAD3* genes, have been deposited in Decipher and the Database of Genomic Variants [48]. With the exception of one individual with hypospadias, none of these patients presented with BEEC, thus rendering a contribution of these genes to disease formation unlikely.

## Conclusions

Available data suggest that disease causing CNVs other than duplications of chromosomal region 22q11.21 are a rare cause of BEEC. Around 98.5 % of cases with BEEC are isolated, and yet many of the described CNVs in this study and by others are inherited from a supposedly healthy parent. This argues either, that non-penetrance is extremely common, or that the CNVs detected are unrelated. Further research is warranted to determine the role of the presently identified CNVs in BEEC etiology. Some of these rare inherited CNVs might at least constitute modifiers or contributors in a multifactorial mode of inheritance.

## Abbreviations

BEEC: bladder exstrophy-epispadias complex
CBE: classic bladder exstrophy
CE: cloacal exstrophy
CNV: copy number variation
Kb: kilobase pairs
MAF: minor allele frequency
Mb: megabase pairs
PCR: polymerase chain reaction
qPCR: quantitative PCR
SNP: single nucleotide polymorphism

## References

[1] Husmann DA, Vandersteen DR, Gearhart JP, Mathews R (1999). Anatomy of the cloacal exstrophy. The exstrophy-epispadias complex.

[2] Hurst JA, Firth HV, Hall JG (2012). Anterior abdominal wall defects. Oxford desk reference. Clinical genetics.

[3] Mahfuz I, Darling T, Wilkins S, White S, Cheng W (2013). New insights into the pathogenesis of bladder exstrophy-epispadias complex. J Pediatr Urol.

[4] Sifel C, Correa A, Amar E, Bakker MK, Bermejo-Sánchez E, Bianca S (2011). Bladder exstrophy: An epidemiologic study from the International Clearinghouse for Birth Defects Surveillance and Research, and an overview of the literature. Am J Med Genet C Semin Med Genet.

[5] Hurwitz RS, Manzon GA, Ransley PG, Stephens FD (1987). Cloacal exstrophy: a report of 34 cases. J Urol.

[6] Wiesel A, Queisser-Luft A, Clementi M, Bianca S, Stoll C, EUROSCAN Study Group (2005). Prenatal detection of congenital renal malformations by fetal ultrasonographic examination: an analysis of 709,030 births in 12 European countries. Eur J Med Genet.

[7] Ebert AK, Reutter H, Ludwig M, Rösch WH (2009). The exstrophy-epispadias complex. Orphanet J Rare Dis.

[8] Boyadjiev SA, Dodson JL, Radford CL, Ashrafi GH, Beaty TH, Mathews RI (2004). Clinical and molecular characterization of the bladder exstrophy-epispadias complex: analysis of 232 families. BJU Int.

[9] Gambhir L, Holler T, Muller M, Schott G, Vogt H, Detlefsen B (2008). Epidemiological survey of 214 families with bladder exstrophy-epispadias complex. J Urol.

[10] Reutter H, Draaken M, Pennimpede T, Wittler L, Brockschmidt FF, Ebert AK (2014). Genome-wide association study and mouse expression data identify a highly conserved 32 kb intergenic region between WNT3 and WNT9b as possible susceptibiliy locus for isolated classic exstrophy of the bladder. Hum Mol Genet.

[11] Draaken M, Knapp M, Pennimpede T, Schmidt JM, Ebert AK, Rösch W (2015). Genome-wide association study and meta-analysis identify ISL1 as genome-wide significant susceptibility gene for bladder exstrophy. PLoS Genet.

[12] Tannour-Louet M, Han S, Corbett ST (2010). Identification of de novo copy number variants associated with human disorders of sexual development. PLoS ONE.

[13] El-Hattab AW, Skorupski JC, Hsieh MH, Breman AM, Patel A, Cheung SW, Craigen WJ (2010). OEIS complex associated with chromosome 1p36 deletion: A case report and review. Am J Med Genet A.

[14] Rotmensch S, Liberati M, Luo JS, Tallini G, Mahoney MJ, Hobbins JC (1991). Prenatal diagnosis of a fetus with terminal deletion of chromosome 1 (q41). Prenat Diagn.

[15] Zaki MS, Gillesseen-Kaesbach G, Vater I, Caliebe A, Siebert R, Kamel AK (2012). Bladder exstrophy and extreme genital anomaly in a patient with pure terminal 1q deletion: Expansion of phenotypic spectrum. Eur J Med Genet.

[16] Jorgez CJ, Rosenfeld JA, Wilken NR, Vangapandu HV, Sahin A, Pham D (2014). Genitourinary defects associated with genomic deletions in 2p15 encompassing OTX1. PLoS ONE.

[17] Kosaki R, Fukuhara Y, Kosuga M, Okuyama T, Kawashima N, Honna T (2005). OEIS complex with del (3) (q12.2q13.2). Am J Med Genet A.

[18] Nicholls G, Duffy PG (1998). Anatomical correction of the exstrophy-epispadias complex: analysis of 34 patients. Br J Urol.

[19] Battaglia A, Carey JC, Cederholm P, Viskochil DH, Brothman AR, Galasso C (1999). Natural history of Wolf-Hirschhorn syndrome: experience with 15 cases. Pediatrics.

[20] Chipail A, Constantinescu V, Covic M, Angheloni T (1976). Phenotypic and cytogenetic analysis of an unusual malformative syndrome (trisomy 9 p+). Rev Pediatr Obstet Ginecol Pediatr.

[21] Thauvin-Robinet C, Faivre L, Cusin V, Khau Van Kien P, Callier P, Parker KL (2004). Cloacal exstrophy in an infant with 9q34.1-qter deletion resulting from a de novo unbalanced translocation between chromosome 9q and Yq. Am J Med Genet A.

[22] Draaken M, Mughal SS, Pennimpede T (2013). Isolated bladder exstrophy associated with a de novo 0.9 Mb microduplication on chromosome 19p13.12.. Birth Defects Res A Clin Mol Teratol.

[23] Draaken M, Reutter H, Schramm C, Bartels E, Boemers TM, Ebert AK (2010). Microduplications at 22q11.21 are associated with non-syndromic classic bladder exstrophy. Eur J Med Genet.

[24] Draaken M, Baudisch F, Timmermann B, Kuhl H, Kerick M, Proske J (2014). Classic bladder exstrophy: Frequent 22q11.21 duplications and definition of a 414 kb phenocritical region. Birth Defects Res A Clin Mol Teratol.

[25] Lundin J, Soderhäll C, Lundén L, Hammarsjö A, White I, Schoumans J (2010). 22q11.2 microduplication in two patients with bladder exstrophy and hearing impairment. Eur J Med Genet.

[26] Pierquin G, Uwineza A (2012). 22q11.2 microduplication in a patient with bladder exstrophy and delayed psychomotor development. Eur J Hum Genet.

[27] Soderhäll C, Lundin J, Lagerstedt-Robinson K, Grigelioniene G, Lackgren G, Clementson Kockum C, Nordenskjöld A (2014). A case with bladder exstrophy and unbalanced X chromosome rearrangement. Eur J Pediatr Surg.

[28] Vlangos CN, Siuniak A, Ackley T, van Bokhoven H, Veltman J, Iyer R (2011). Comprehensive genetic analysis of OEIS complex reveals no evidence for a recurrent microdeletion or duplication. Am J Med Genet A.

[29] Schmermund A, Möhlenkamp S, Stang A, Grönemeyer D, Seibel R, Hirche H (2002). Assessment of clinically silent atherosclerotic disease and established and novel risk factors for predicting myocardial infarction and cardiac death in healthy middleaged subjects: rationale and design of the Heinz Nixdorf RECALL Study. Risk factors, evaluation of coronary calcium and lifestyle. Am Heart J.

[30] Colella S, Yau C, Taylor JM, Mirza G, Butler H, Clouston P (2007). An Objective Bayes Hidden-Markov Model to detect and accurately map copy number variation using SNP genotyping data. Nucleic Acids Res.

[31] Meyer LR, Zweig AS, Hinrichs AS, Karolchik D, Kuhn RM, Wong M (2013). The UCSC Genome Browser database: extensions and updates 2013. Nucleic Acids Res.

[32] Draaken M, Giesen CA, Kesselheim AL, Jabs R, Aretz S, Kugaudo M (2011). Maternal de novo triple mosaicism for two single OCRL nucleotide substitutions (c.1736A > T, c.1736A > G) in a Lowe syndrome family. Hum Genet.

[33] Ludwig M, Ching B, Reutter H, Boyadjiev SA (2009). Bladder exstrophy-epispadias complex. Birth Defects Res Part A Clin Mol Teratol.

[34] Froyen G, van Esch H, Bauters M, Hollanders K, Frints SG, Vermeesch JR (2007). Detection of genomic copy number changes in patients with idiopathic mental retardation by high-resolution X-array-CGH: Important role for increased gene dosage of XLMR genes. Hum Mutat.

[35] Wieland I, Jakkubiczka S, Muschke P, Cohen M, Thiele H, Gerlach KL, Adams RH, Wieacker P (2004). Mutations of the ephrin-B1 gene cause craniofrontonasal syndrome. Am J Hum Genet.

[36] Babbs C, Stewart HS, Williams LJ, Connell L, Goriely A, Twigg SRF (2011). Duplication of the EFNB1 gene in familial hypertelorism: Imbalance in Ephrin-B1 expression and abnormal phenotypes in humans and mice. Hum Mutat.

[37] Neidlich JA, Whitaker LA, Natowicz M, McDonald DM, Schnur R, Zackai EH (1988). Aglossia with congenital absence of the mandibular rami and other craniofacial abnormalities. Am J Med Genet.

[38] Robin NH, Neidich JA, Bason LD, Whitaker LA, McDonald-McGinn D, Hunter J, Snyder HM, Zackai EH (1996). Frontonasal malformation and cloacal exstrophy: A previously unreported asociation. Am J Med Genet.

[39] Visel A, Thaller C, Eichele G (2004). GenePaint.org: an atlas of gene expression patterns in the mouse embryo. Nucleic Acids Res.

[40] Dravis C, Yokoyama N, Chumley MJ, Cowan CA, Silvany RE, Shay J, Baker LA, Henkemeyer M (2004). Bidirectional signaling mediated by ephrin-B2 and EphB2 controls urorectal development. Dev Biol.

[41] Walczak-Sztulpa J, Wisniewska M, Latos-Bielenska A, Linné M, Kelbova C, Belitz B (2008). Chromosome deletions in 13q33-34: Report of four patients and review of the literature. Am J Med Genet A.

[42] Pinto D, Marshall C, Feuk L, Scherer SW (2007). Copy-number variation in control population cohorts. Hum Mol Genet.

[43] Redon R, Ishikawa S, Fitch KR, Feuk L, Perry GH, Andrews TD (2006). Global variation in copy number in the human genome. Nature.

[44] Zogopoulos G, Ha KC, Naqib F, Moore S, Kim H, Montpetit A (2007). Germ-line DNA copy number variation frequencies in a large North American population. Hum Genet.

[45] Giannikou K, Fryssira H, Oikonomakis V, Syrmou A, Kosma K, Tzetis M, Kitsiou-Tzeli S, Kanavakis E (2012). Further delineation of novel 1p36 rearrangements by array-CGH analysis: Narrowing the breakpoints and clarifying the “extended” phenotype. Gene.

[46] Goller T, Seibold UK, Kremmer E, Voos W, Kolanus W (2013). Atad3 function is essential for early post-implantation development in the mouse. PLoS Genet.

[47] Li S, Lamarche F, Charton R, Delphin C, Gires O, Hubstenberger A, Schlattner U, Rousseau D (2014). Expression analysis of ATAD3 isoforms in rodent and human cell lines and tissues. Gene.

[48] MacDonald JR, Ziman R, Yuen RK, Feuk L, Scherer SW (2013). The database of genomic variants: a curated collection of structural variation in the human genome. Nucleic Acids Res.

